# Evaluation of skeletal muscle function in male rats with doxorubicin-induced myopathy following various exercise techniques: the significant role of glucose transporter 4

**DOI:** 10.1007/s00424-024-02922-3

**Published:** 2024-02-17

**Authors:** Eman Osama, Effat Khowailed, L Rashed, A. Fawzy, Rokia Mohamad Hassan, Inas Harb, Muhammad Maher

**Affiliations:** 1 Department of Medical Physiology, Faculty of Medicine, Cairo University, Giza, Egypt; 2 Department of Medical Biochemistry, Faculty of Medicine, Cairo University, Giza, Egypt; 3 Department of Medical Histology, Faculty of Medicine, Cairo University, Giza, Egypt; 4 Department of Medical Pharmacology, Faculty of Medicine, Cairo University, Giza, Egypt

**Keywords:** Doxorubicin, Muscle atrophy, Fatigue, Myopathy, Exercise, Insulin resistance

## Abstract

A common anthracycline antibiotic used to treat cancer patients is doxorubicin (DOX). One of the effects of DOX therapy is skeletal muscle fatigue. Our goal in this research was to study the beneficial effect of exercise on DOX-induced damaged muscle fibers and compare the effect of different exercise strategies (prophylactic, post- toxicity and combined) on DOX toxicity. Five groups were created from 40 male rats: group I, control group; group II, DOX was administered intraperitoneally for 2 weeks over 6 equal injections (each 2.5 mg/kg); group III, rats trained for 3 weeks before DOX; group IV, rats trained for 8 weeks after DOX; and group V, rats were trained for 3 weeks before DOX followed by 8 weeks after. Measures of oxidative damage (H_2_O_2_, catalase), inflammation (TNF-α), and glucose transporter 4 (GLUT4) expression on skeletal muscle were assessed. Also, Homeostatic Model Assessment for Insulin Resistance (HOMA-IR) was estimated. Skeletal performance was evaluated by contraction time (CT), half relaxation time (1/2 RT), and force-frequency relationship by the end of this research. The current study demonstrated a detrimental effect of DOX on skeletal performance as evidenced by a significant increase in CT and 1/2 RT compared to control; in addition, H_2_O_2_, TNF-α, and HOMA-IR were significantly increased with a significant decrease in GLUT4 expression and catalase activity. Combined exercise therapy showed a remarkable improvement in skeletal muscle performance, compared to DOX, CT, and 1/2 RT which were significantly decreased; H_2_O_2_ and TNF-α were significantly decreased unlike catalase antioxidant activity that significantly increased; in addition, skeletal muscle glucose metabolism was significantly improved as GLUT4 expression significantly increased and HOMA-IR was significantly decreased. Exercise therapy showed significant improvement in all measured parameters relative to DOX. However, combined exercise therapy showed the best improvement relative to both pre-exercise and post-exercise groups.

## Introduction

For many years, oncologists have been extremely concerned about the cardiac toxicity of the chemotherapy drug doxorubicin (DOX). Although the primary consequence of DOX is cardiotoxicity, having a bad quality of life is also caused by fatigue and skeletal muscle atrophy following DOX treatment [1]. Oxidative stress induction is the main mediator of DOX toxicity. There are numerous mechanisms for DOX to generate reactive oxygen species (ROS). Lipid peroxidation can be started by superoxide and its breakdown products, which can harm healthy tissue [2]. This increase in oxidation additionally induces tissue inflammation and ruins proper tissue apoptosis which probably causes more tissue damage.

As a result of several pharmacological treatments, including DOX therapy, it has been demonstrated that the insulin sensitivity of skeletal muscle is decreasing. About 75% of the absorption of circulating glucose occurs in skeletal muscle. Insulin resistance encourages muscle atrophy, which lowers performance and gives off physically crippling tiredness [3]. Therefore, a potential link between DOX use and insulin resistance as well as the part played by skeletal muscle in the process is needed to be looked into.

Exercise training has been shown to have cytoprotective benefits against DOX toxicity and may be an essential weapon in the defense against toxic consequences [2]. Ji and Zhang [4] hypothesized that moderate-intensity exercise has antioxidant and anti-inflammatory properties because it affects many redox-sensitive signal transduction pathways. It also appears that exercise training has antiapoptotic effects in hypertensive hearts by inhibiting two major apoptotic pathways, as shown by decreases in Fas receptor or mitochondria-dependent apoptotic proteins which activate further apoptotic factors [5].

In light of these facts, our goal was to ascertain the positive effects of exercise as a non-pharmacologic strategy for treating DOX-induced skeletal myopathy. In addition, we compared the effect of different exercise strategies (prophylactic, post-toxicity, and combined exercise by training before and after DOX exposure) on DOX toxicity to clarify if exercising on a routine basis will be beneficial upon exposure to DOX therapy.

## Materials and methods

### Study design

The Cairo University Animal House purchased and cared for the animals. Physiology, Biochemistry, Pharmacology, and Histology departments at Cairo University’s Faculty of Medicine participated in the study.

Forty male Wistar rats, 8 weeks of age weighing about 250 g, were used for this experiment. Animals were left for a few days to acclimatize to ordinary environmental living conditions, as regards humidity, temperature, and dark/light cycles. They were housed in wire-mesh cages (5 rats in each) and had free access to food and water before starting the experimental procedure.

Experimental animal methods and animal operations were authorized by Cairo University’s Institutional Animal Care and Use Committee (IACUC) (approval number: CU-III-F-49–20).

### Animal grouping

Rats were divided into 5 research groups as follows.

***Group I: control group (n*** **=** ***8)***

Rats were intraperitoneally administered 0.9% NaC1 solution in a volume comparable to the DOX administered to group II.

***Group II: DOX group (n*** **=** ***8)***

Six identical intraperitoneal DOX doses (2.5 mg/kg/injection) were given to rats over 2 weeks, totaling 15 mg/kg [6].

***Group III: Pre-exercise group (pre-E) (n*** **=** ***8)***

Rats were subjected to a pre-exercise protocol. They were trained (swimming) for 3 weeks before DOX administration [7].

***Group IV: Post exercise group (post-E group) (n*** **=** ***8)***

Rats were subjected to a post-exercise protocol. They were trained for 8 weeks after DOX [8].

***Group V: Combined exercise group (CE) (n*** **=** ***8)***

Rats were subjected to a combined exercise protocol. They were trained for 3 weeks before DOX administration and then 8 weeks after DOX.

### Chemicals

1. A vial of 25 ml of adriamycin (doxorubicin HCL), a medication made by Hikma Specialized Pharmaceuticals in Egypt, includes 2 mg of doxorubicin HCl/ ml.
2. Urethane powder a product of (Sigma-Tec, Egypt).

### Drug preparation and administration

Adriamycin vial was diluted with an equal amount of saline, so each ml of the solution contains 1 mg of doxorubicin HCl and then given intraperitoneal in a dose of 2.5 mg/kg.

### Physical exercise protocol

Rats were acclimated to warm water (30–32 °C), 5 days per week, for 2 weeks before the regimen of physical training began. The aim of adaptation was to reduce animal stress. Rats were placed in shallow water for 10 min for 3 days. The water depth was then increased gradually. By the 4th day, animals swam for 5 min in deep water. The length of time was increased by 10 min each day until the 10th day of adaptation, the 50-L containers used for physical exercise had water that was maintained between 30 and 32 degrees Celsius. According to the American Physiological Society’s advice, the swimming protocol was followed:

## Pre-treatment swimming exercise

For 3 weeks, the exercising rats underwent daily 90-min swimming workouts 5 days a week. The initial swimming session lasted 20 to 30 min, and the duration was gradually extended by 30 min each time, reaching 90 min maintained until the completion of the training period [7].

## Post-treatment swimming exercise

The training rats underwent a 60-min daily swimming workout, 5 days per week, for 8 weeks. The rats swam for 20 min for the first 7 days of training, and then for 30 min, 40 min, and eventually 50 min on the 4th week. The animals engaged in continuous 60-min exercise from the end of the fourth week until the program’s conclusion (after 8 full weeks) [8].

## Combined exercise protocol

Rats in this group were trained by swimming before and after DOX exposure using the same protocol that was applied separately at the pre-treatment and post-treatment protocol.

### Skeletal performance assessment using power lab

Animals were anesthetized with urethane 0.7 mg/kg. To assess the contractile characteristics of the gastrocnemius muscle in situ, each rat was laterally positioned on a heating pad that was kept at 37 °C. The left gastrocnemius muscle and supplying sciatic nerve were surgically exposed and irrigated with saline solution. The rats left hind limbs were securely immobilized. With 4.0 silk, the muscle’s distal tendon was connected to an isometric force transducer. An MLA0320 nerve-stimulating electrode (AD Instruments) was used to contact the exposed sciatic nerve [9]. Contractions elicited at 2-min intervals using stimulus frequencies of 1, 15, 30, 60, and 120 Hz were used to assess the force-frequency relationship [10] Measurements also included single-twitch contraction time (CT) and half-relaxation time (1/2 RT).

Using Power Lab 4/30 Dual Bio/ Stim, Lab Chart, AD Instruments, all data were gathered.

### Samples collection and tissue harvest

Following the evaluation of skeletal muscle performance, at the end of the study protocol:

- A capillary tube was used to collect blood samples from the retro-bulbar plexus into 10 ml Eppendorf tubes for biochemical analysis.
- Animals were killed by cervical dislocation, and gastrocnemius muscles were removed for the following:

- Biochemical assessment
- Histological assessment

### Biochemical assessment

HOMA-IR was calculated using blood samples for the measurement of fasting glucose and insulin. The measures for oxidative stress [hydrogen peroxide (H_2_O_2_), catalase activity], inflammatory parameters [tumor necrosis factor-alpha (TNF-α)], GLUT4, and parameters for identifying apoptosis [caspase 3] were all measured in the muscles.

- Prior to dissection, skeletal muscle tissues were perfused with a PBS (phosphate buffered saline) solution, pH 7.4 containing 0.16 mg /ml heparin to remove any red blood cells and clots and then homogenized in 5–10 ml cold buffer (50 mM potassium phosphate) per gram tissue and Centrifuged at 10,000xg for 15 min at 4 °C.
- Following the previous, the supernatant was removed for assay and stored on ice.

#### Determination of fasting serum glucose level

“Diamond Diagnostics” provided the kits used for this approach. The interassay coefficient of variation for glucagon was 6.0%, and the intrasssay coefficient was 2.6%.

#### Determination of fasting serum insulin level

By employing the rat insulin ELISA kits, insulin concentrations were determined in serum samples that had been frozen and thawed before. The interassay coefficient of variation for glucagon was 9.8%, and the intrasssay coefficient was 1.5%.

#### Estimation of Homeostasis Model Assessment Insulin Resistance index (HOMA-IR)

Serum insulin (IU/L) multiplied by serum glucose (mmol/L) divided by 22.5 was used to create the HOMA-IR index: Insulin resistance is diagnosed when HOMA-IR is more than 4.0 [11].

#### Catalase activity and H_2_O_2_ levels in tissues estimated using a colorimetric method

Rat calorimetric kit Catalog No. HP 25 30, CA 25 16 were used to measure tissue H_2_O_2_ and catalase activity, respectively. The process was carried out by the manufacturer’s guidelines.

Hydrogen peroxide measurement using calorimetric kit Catalog No. HP 25 30 depends on the following principle: In the presence of peroxidase ( HRP), H_2_O_2_ reacts with 3,5-dichloro-2-hydroxybenzensulfonic ( DHBS) acid and 4-aminophenazone ( AAP) to form a chromophore as shown in this reaction:

$$2 {{\text{H}}}_{2}{{\text{O}}}_{2}+{\text{DHBS}}+\mathrm{AAP }\stackrel{{\text{HRP}}}{\to }\mathrm{Quinoneimine Dye }+4{{\text{H}}}_{2}{\text{O}}$$

#### TNF-α level estimation in tissues by ELISA

Rat TNF-α ELISA Kit, E-EL-R2856, utilized for in vitro quantitative assessment of rat TNF-concentrations in tissue homogenates. With detection Range 15.63–1000 pg/mL. In the intraassay precision (precision within an assay), 3 samples with low, mid-range, and high-level rat TNF-α were tested 20 times on one plate, respectively. In the interassay precision (precision between assays), 3 samples with low, mid-range, and high-level rat TNF-α were tested on 3 different plates, 20 replicates in each plate, respectively. The interassay coefficient of variation for glucagon was 2.4%, and the intrasssay coefficient was 0.07%.

The Rat TNF-α ELISA Kit utilizes the quantitative sandwich enzyme immunoassay technique. These kits come with micro-ELISA plates that have been pre-coated with an antibody that is specific to rat TNF-α. The micro-ELISA plate wells are filled with samples (or standards) and the specific antibody mixture.

#### Quantitative real-time PCR analysis of GLUT4 gene expression

Total RNA was isolated using Qiagen tissue extraction kit (Qiagen, USA) according to instructions of manufacture. The total RNA (1µg) was used for complementary DNA (cDNA) conversion using high-capacity cDNA reverse transcription kit (Fermentas, USA). Moloney murine leukemia virus (MMLV) reverse transcriptase was used for synthesis of cDNA from RNA. Human placental ribonuclease inhibitor (HPRI) was used for inhibition of RNase activity. Real-time qPCR amplification and analysis were performed using an Applied Biosystem with software version 3.1 (StepOne™, USA). The reaction contained SYBR Green Master Mix (Applied Biosystems), gene-specific primer pairs which were shown in the Table 1 and were designed with Gene Runner Software (Hasting Software, Inc., Hasting, NY) from RNA sequences from the gene bank. All the primer sets had a calculated annealing temperature of 60°. Amplification conditions were 2 min at 50°, 10 min at 95°, and 40 cycles of denaturation for 15 s and annealing/extension at 60° for 10 min. The relative expression of the studied genes was calculated according to Applied Bio system software using the comparative threshold cycle method. All values were normalized to the B-actin expression gene as the house keeping (reference) gene.

**Table 1:** Primers sequence of studied genes

Gene	Primer sequence
GLUT4	**Forward primer:** 5′-GGGCCTGCCCGAAAGAGTCT-3′ **Reverse primer:** 5′-AGGCTGGCTGTTCCACCCCA-3′
βeta actin	**Forward primer:** 5′-TGTTTGAGACCTTCAACACC-3′ **Reverse primer:** 5′-CGCTCATTGCCGATAGTGAT-3′

### Histological and immunohistochemical studies

Left gastrocnemius muscle samples were excised, washed in saline, and fixed for 48 h in 10% formol saline and then processed separately in 5–7 μm paraffin sections. Sections were submitted to: hematoxylin and eosin stain [12].

A. Reagents used for staining

1. Mayer’s hemalum

- Hematoxylin [1.0 gm], aluminum sulphate [50 gm], citric acid [1 gm], sodium iodate [0.1gm], and water [750 ml]

2 Eosin

- Eosin yellowish [2.5 gm], water [495 ml], and glacial acetic acid [0.5 ml]

3 Acid alcohol

- Alcohol 95% [500 ml] and concentrated hydrochloric acid [5 ml]

B. Steps of staining

1. Paraffin sections were de-waxed and hydrated then stained in Mayer’s hemalum for 1–5 min (overstained sections can be easily differentiated by agitating in acid alcohol and then washed in running tap water). After that, sections were washed in running tap water for 2–3 min or until the sections turned blue.
2. Sections were immersed in eosin for 30 s (and differentiated in acid alcohol), washed in running tap water for 30 s and then dehydrated in ascending grades of ethanol (70%, 95%, 100%), 5 min each.
3. Finally, slides were cleared in xylene and then mounted in Canada balsam.

### Statistical methods

Data coding and entry were carried out using SPSS version 28 (Statistical Package for the Social Sciences) (IBM Corp., Armonk, NY, USA). Data were compiled using mean and standard deviation. Analysis of variance (ANOVA) with multiple comparisons Tukey post hoc test was used to compare the groups [13]. Statistics were considered significant for *P* values under 0.05.

## Results

### Effect on measured skeletal muscle parameters

As shown in Table 2, H2O2 as a marker of oxidative stress was significantly increased in DOX group compared to control group. Both post-E and CE groups showed a significant decrease in H_2_O_2_ relative to DOX. Catalase activity showed a significant increase in CE group relative to DOX, pre-E, and post-E groups. All exercise-trained groups showed obvious improvement in TNF-α compared to both DOX and control groups with a significant decrease in CE group relative to other trained groups. Additionally, both CE and post-E groups showed a significant increase in GLUT4 expression compared to pre-E group.

**Table 2:** Measured skeletal muscle parameters

	Control	DOX	Pre-E	Post-E	CE
H_2_O_2_ mM/g	**19.08 ± 3.78**	77.99 ± 7.5^*^	72.39 ± 5.93^*^	44.06 ± 8.38^*#@^	**27.73 ± 4.99**^**#@&**^
catalase U/g	**200.69 ± 6.89**	97.01 ± 7.52^*^	136.01 ± 6.15^*#^	170.4 ± 5.04^*#@^	**193.75 ± 5.31**^**#@&**^
TNF- α pg/g	**61.13 ± 5.65**	206.53 ± 5.59^*^	138.56 ± 8.28^*#^	109.65 ± 5.22^*#@^	98.65 ± 6.31^*#@&^
GLUT4	**1.02 ± 0.02**	0.19 ± 0.05^*^	0.42 ± 0.08^*#^	0.73 ± 0.08^*#@^	0.81 ± 0.06^*#@^

Values are presented as mean ± SD

^*^Statistically significant compared to corresponding value in control group (*P* < 0.05)

^#^Statistically significant compared to corresponding value in DOX group (*P* < 0.05)

^@^Statistically significant compared to corresponding value in pre-E group (*P* < 0.05)

^&^Statistically significant compared to corresponding value in post-E group (*P* < 0.05)

### Effect on serum glucose level and insulin resistance

Insulin resistance was significantly higher in the DOX group compared to the control group. Different exercise protocols markedly reduced insulin resistance. CE and post-E groups showed better improvement in blood tests relative to pre-E group as shown in Table 3.

**Table 3:** Serum glucose level and insulin resistance in studied groups

	Control	DOX	Pre-E	Post-E	CE
Fasting glucose mmol/L	**5.04 ± 0.33**	16.71 ± 1.28^*^	13.22 ± 2.13^*#^	8.04 ± 1.01^*#@^	**6.51 ± 1.14**^**#@**^
Fasting insulin μIU/L	**9.44 ± 0.92**	21.49 ± 2^*^	16.11 ± 2.17^*#^	**9.97 ± 1.52**^**#@**^	**9.51 ± 1.45**^**#@**^
IR	**2.11 ± 0.25**	16 ± 2.31^*^	9.48 ± 2.16^*#^	**3.55 ± 0.66**^**#@**^	**2.76 ± 0.69**^**#@**^

Values are presented as mean ± SD

^*^Statistically significant compared to corresponding value in control group (*P* < 0.05)

^#^Statistically significant compared to corresponding value in DOX group (*P* < 0.05)

^@^Statistically significant compared to corresponding value in pre-E (*P* < 0.05)

^&^Statistically significant compared to corresponding value in post-E (*P* < 0.05)

### Effect on skeletal performance

**Fig. 1:**
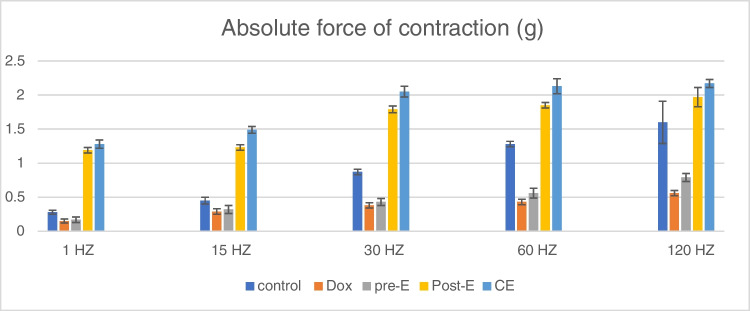
Clustered column showing force generated at 1, 15, 30, 60, and 120 Hz in different groups

As Table 4 shows, CT and 1/2 RT were significantly decreased in CE group relative to pre-E but showed no significant decrease relative to post-E group. Skeletal muscle force of contraction was highly reduced by DOX treatment, and exercise training was effective in restoring muscle power. At all frequencies, CE showed the best improvement relative to other trained groups (Table 5 and Fig. 1).

**Table 4:** CT and 1/2 RT in all groups

	Control	DOX	Pre-E	Post-E	CE
CT ms	**23.75 ± 11.88**	83.75 ± 31.14^*^	**47.50 ± 16.69**^**#**^	**20.00 ± 7.56**^**#@**^	**15.88 ± 4.02 **^**#@**^
1/2 RT ms	**72.50 ± 5.98**	196.25 ± 9.16^*^	93.75 ± 14.58^*#^	42.50 ± 7.07^*#@^	30.63 ± 7.29^*#@^

Values are presented as mean ± SD

^*^Statistically significant compared to corresponding value in control group (*P* < 0.05)

^#^Statistically significant compared to corresponding value in DOX group (*P* < 0.05)

^@^Statistically significant compared to corresponding value in pre-E group (*P* < 0.05)

^&^Statistically significant compared to corresponding value in post-E group (*P* < 0.05)

**Table 5:** Force generated at different frequencies in all groups

	Control	DOX	Pre-E	Post-E	CE
Force (g) at 1 HZ	**0.28 ± 0.03**	0.15 ± 0.03^*^	0.17 ± 0.04^*^	1.19 ± 0.04^*#@^	1.28 ± 0.06^*#@&^
force (g) at 15 HZ	**0.45 ± 0.05**	0.29 ± 0.04^*^	0.32 ± 0.06^*^	1.23 ± 0.04^*#@^	1.49 ± 0.05^*#@&^
force (g) at 30 HZ	**0.87 ± 0.04**	0.38 ± 0.04^*^	0.43 ± 0.05^*^	1.79 ± 0.05^*#@^	2.05 ± 0.08^*#@&^
force (g) at 60 HZ	**1.28 ± 0.04**	0.43 ± 0.04^*^	0.56 ± 0.07^*#^	1.85 ± 0.04^*#@^	2.13 ± 0.11^*#@&^
force (g) at 120 HZ	**1.6 ± 0.31**	0.56 ± 0.04^*^	0.79 ± 0.06^*#^	1.97 ± 0.14^*#@^	2.17 ± 0.06^*#@^

Values are presented as mean ± SD

^*^Statistically significant compared to corresponding value in control group (*P* < 0.05)

^#^Sstatistically significant compared to corresponding value in DOX group (*P* < 0.05)

^@^Statistically significant compared to corresponding value in pre-E group (*P* < 0.05)

^&^Statistically significant compared to corresponding value in post-E group (*P* < 0.05)

To make the difference between the exercise groups more obvious, we measured % of change of the results of measured parameters relative to DOX results as shown in Table 6.

**Table 6:** % of change of exercise-trained groups measured parameters values relative to DOX group

	Pre-E	Post-E	CE
CT	** − 43.28**	** − 76.12**	** − 81.04**
1/2 RT	** − 52.23**	** − 78.34**	** − 84.39**
H_2_O_2_	** − 7.18**	** − 43.51**	** − 64.44**
Catalase	**40.20**	**75.65**	**99.72**
TNF- α	** − 32.91**	** − 46.91**	** − 52.23**
GLUT4	**121.05**	**284.21**	**326.32**
Fasting glucose	** − 20.89**	** − 51.89**	** − 61.04**
Fasting insulin	** − 25.03**	** − 53.61**	** − 55.75**
HOMA-IR	** − 40.75**	** − 77.81**	** − 82.75**

### Histopathological examination (Fig. 2)

**Fig. 2:**
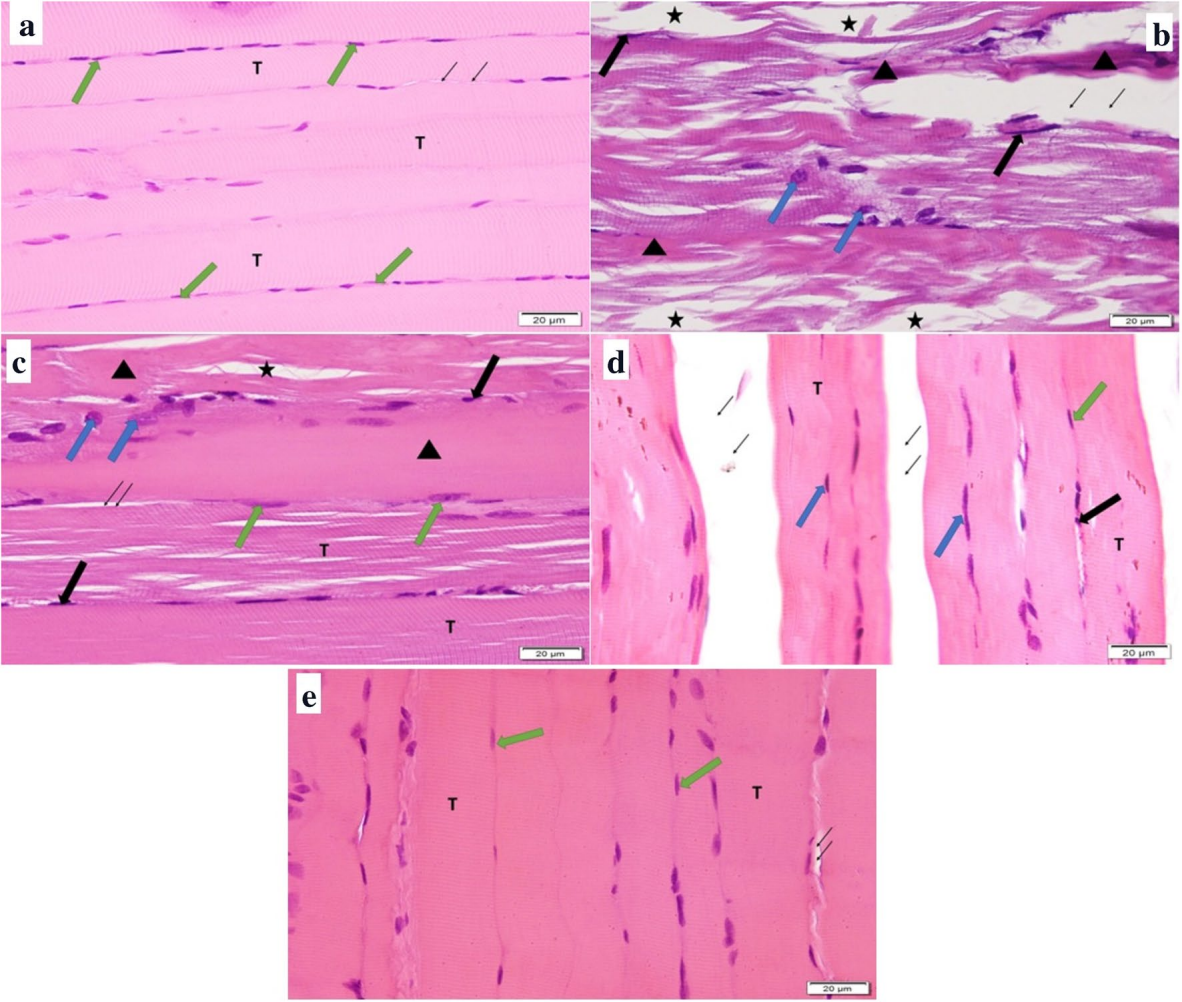
Photomicrographs of a control, b DOX, c pre-E, d post-E, and e CE groups (H&E, 400 × scale bar = 20 μm). Green arrow: oval peripheral vesicular nuclei. T: transverse striations. Thin arrows: endomysium. Triangles: sarcoplasm striation. Black arrows: darkly stained pyknotic nuclei. Stars: loss of myofibrils. Blue arrows: centrally located nuclei

Skeletal muscle longitudinal sections of the control group revealed normal histological structure of skeletal muscle fibers which appeared cylindrical, non-branching, non-anastomosing, and parallel to each other. Sarcoplasm skeletal muscle fibers appeared acidophilic with clear transverse striations and multiple oval vesicular nuclei which were peripherally located under the sarcolemma.

Longitudinal sections of DOX group revealed degeneration and atrophy as muscle fibers appeared disrupted with deep acidophilic sarcoplasm, loss of transverse striations, loss of myofibrils, dark pyknotic nuclei, and wide separation of muscle fibers by endomysium. Centrally located nuclei were also observed.

Pre-exercise training only revealed mild improvement in skeletal muscle structure as some muscle fibers appeared with deep acidophilic sarcoplasm, loss of transverse striations, focal loss of myofibrils, and darkly stained pyknotic nuclei, while others appeared with clear transverse striations and vesicular nuclei. Narrow endomysium and some centrally located nuclei were observed. On the other hand, post-exercise group showed moderate improvement in cardiac muscle structure as most of skeletal muscle fibers appeared well-organized with acidophilic striated sarcoplasm and multiple oval vesicular peripheral nuclei. Some darkly stained pyknotic nuclei were noticed and others were centrally located. Wide endomysium was also observed.

CE therapy restored normal muscle structure as fibers appeared normally well-organized parallel to each other with acidophilic sarcoplasm, clear transverse striations, and multiple oval vesicular peripherally located nuclei. Narrow endomysium separating muscle fibers was observed.

## Discussion

It has been demonstrated that DOX causes toxic effects on skeletal muscle in addition to cardiotoxicity [14]. Due to the potential for life-threatening cardiotoxicity, its effects on cardiomyocytes have drawn the most attention. Our data demonstrated poorer skeletal muscle performance in DOX rats compared to the control group by a large rise in CT and 1/2 RT with a significant decrease in force generated at various frequencies. This data supported a study done by Ge et al. [6] which found that rats treated with DOX had impaired diaphragmatic contractility.

Numerous studies suggest that considerable ROS generation [15], decreased antioxidant enzyme activity [16], inflammation, and protease activation [17] may all play a role in skeletal dysfunction.

From a biological perspective, a number of enzymes such as xanthine oxidases, NADPH oxidases, and nitric oxide synthases found in subcellular components like mitochondria, the sarcoplasmic reticulum, and the cytoplasm are sources of reactive oxygen species. These enzymes have the ability to break down DOX into the semiquinone intermediate, which can quickly break down oxygen into superoxide (O2•–) [18]. Together, doxorubicin and iron form a complex that releases superoxide radical. Hydrogen peroxide is changed into hydroxyl radicals, a more potent type of ROS, by this complex. Dox can raise intracellular iron concentrations via a variety of mechanisms, such as ferritin-mediated iron release, ferritin synthesis reduction [19], and increased transferrin receptors on the plasma membrane [20]. Dangerously, a process known as the iron-catalyzed Haber–Weiss reaction can also produce extremely reactive and toxic hydroxyl radicals (OH•) when H_2_O_2_ and O2•–are present [21].

According to Kalyanaraman [22], the main mechanism of DOX toxicity was suggested to be the production of O2•–, H_2_O_2_, and iron-catalyzed hydroxyl radical formation from DOX reduction. Elevated reactive oxygen species (ROS) can trigger cytotoxic signaling, which can result in DNA damage, malfunctioning mitochondria, reduced protein synthesis, and disruption of intracellular calcium homeostasis [23].

The production of excess H_2_O_2_ by the mitochondrial respiratory chain in skeletal muscle was responsible for the repression of mitochondrial respiratory capacity subsequent to DOX treatment [24]. According to Gilliam et al. [10], systemic DOX treatment causes oxidative stress and disruption of redox cycling, which in turn affects myofibrillar order and function. Poly ADP-ribose polymerases (PARPs), which use energy cofactors to cause excess ATP consumption that affects skeletal muscle function, are among the repairing enzymes activated by DNA breaks caused by DOX toxicity [25]. PARP activation also impacts the metabolic state of skeletal muscles through decreased mitochondrial biogenesis and glucose metabolism, which encourage the transition of skeletal muscle fibers from the oxidative to the glycolytic types [26].

In this study, H_2_O_2_ level in the skeletal muscle of DOX rats was substantially higher than those in the control group, but catalase activity was significantly lower. According to Chen et al. [16], ROS accumulation and antioxidant enzyme activity in skeletal muscles of DOX-treated rats were severely impacted.

DOX has been demonstrated to interact directly with skeletal muscle, resulting in structural and functional harm that causes muscle weakening [27]. Dox buildup in skeletal muscle causes modifications, or remodeling in the structural and functional elements of the muscle, impairing muscle performance [28].

Research has demonstrated the importance of factors other than ROS, such as inflammation, in DOX-related toxicity [29]. Hadi et al. [30] demonstrated that the pathophysiology of DOX-induced toxicity includes the activation of the innate immune system subsequent to the release of proinflammatory cytokines.

The imbalance between pro-inflammatory and anti-inflammatory cytokines has also been proposed as a possible mediator of muscular dysfunction. When compared to the control group in the current study, TNF-α levels in the skeletal muscle of DOX rats were considerably higher.

TNF-α upregulation in DOX-treated rats stimulates skeletal muscle ROS generation, which interferes with contractile function [31]. Al-Lamki et al. [32] also demonstrated that TNF-α stimulates NF-κB, which results in subsequent protein degradation and suppression of myogenic differentiation [33]. Doxorubicin-induced increases in TNF-α can result in cytotoxic cascades, intensified oxidants, and muscle weakness, all of which are mediated through TNFR1. Since TNF-α may have an additive oxidant effect, one of the most widely accepted explanation for contractile dysfunction is increased ROS via excess TNF-α [34]. Oxidative stress-sensitive proteins can undergo modifications that impact their ability to communicate and contract. According to Barreiro and Hussain [35], oxidants can alter the structure of contractile proteins and reduce force production when myosin and actin are exposed to them.

It has been speculated that insulin resistance may result in a diminished anabolic response and muscle loss [36]. The activation of NF-κB and proinflammatory cytokines, oxidative stress, and lipid peroxidation caused by DOX reduce insulin sensitivity and damage the insulin signaling pathway.

The current investigation showed that in contrast to control group, DOX group had significantly lower levels of GLUT4 expression in skeletal muscle, significantly higher plasma levels of glucose, insulin, and the HOMA-IR index. In accordance with the prior, de Lima Junior and colleagues [3] highlighted that the suppression of AMPk (AMP-activated protein kinase) signaling in skeletal muscle is what causes the hyperglycemia and insulin resistance caused by DOX. In addition to controlling GLUT4 gene expression, AMPk serves as a crucial energy level sensor in cells. Skeletal muscles’ AMPk signaling appears to be disturbed by DOX-induced oxidative stress [37].

Additionally histological examination of skeletal muscles exposed to DOX also confirmed the toxic effect of DOX on skeletal muscle fibers which definitely will impair their contractile function. Hematoxylin and eosin staining was used to show the muscle fiber damage that results from DOX treatment. Our histological slides show that, in comparison to other groups, the DOX animals had disrupted myofiber ultrastructure. In agreement with Yu et al. [38], DOX treated group showed skeletal muscle fiber atrophy, non-striated sarcoplasm, and darkly pigmented pyknotic nuclei.

One of the well-known issues with cancer patients undergoing DOX therapy is myotoxicity. Therefore, to maintain the efficiency of DOX while minimizing the negative side effects, effective adjuvant therapies are required. We needed to assess exercise treatment in this study to see if it might be used as a technique to prevent muscular dysfunction in DOX-induced myopathy.

According to Huertas et al. [39], endurance exercise training has been demonstrated to lessen the impairment of force output and skeletal muscle atrophy brought on by DOX treatment. This study showed that exercise training enhanced skeletal muscle performance, compared to DOX group, CT, and 1/2 RT which were significantly decreased by − 43.28% and − 52.23% respectively in pre-E group; by − 76.12% and − 78.34% respectively in post-E group; and by − 81.04% and − 84.39% respectively in CE group. All 3 groups showed a significant increase in force of contractions at higher frequencies, while at lower frequencies, only post-E and CE groups showed a significant increase in force of contractions relative to DOX group; pre-E did not significantly improve the force of contraction.

The greatest improvement in skeletal muscle 1/2 RT and force of contraction was seen in post-E and CE groups. Also, 1/2 RT was significantly shorter, and force of contraction was significantly higher in both groups relative to corresponding values in pre-E and even control groups. However, same parameters in pre-E did not show full recovery and were still significantly different than corresponding value in control group. Although CT was improved in pre-E, post-E, and CE groups with no significant difference relative to control group, values in post-E and CE groups were significantly shorter relative to pre-E group.

The therapeutic effects of exercise training against DOX-induced myotoxicity may involve molecular processes that increase antioxidant production, reduce inflammation, or regulate proapoptotic signaling [40, 41].

Regular and consistent physical activity can alleviate the negative effects of oxidative stress caused by DOX. Exercise strengthens the antioxidant defense system by promoting the synthesis of antioxidant enzymes, such as glutathione peroxidase and superoxide dismutase, and reducing lipid peroxidation [42]. It also enhances endothelial function [43] by reducing oxidative stress by balancing reactive oxygen species, which influences nitric oxide bioavailability [44]. Muscle performance is limited, and oxidant production is increased with hard and intense exercise for at least thirty minutes [45]. In contrast to intense training, progressive and gradual training makes it easier for cells to detoxify more ROS, which helps to prevent oxidative stress [46]. Compared to individuals who are not trained, those who exercise on a regular basis show higher levels of mitochondria and lower levels of ROS [47].

In the current study, H_2_O_2_ was greatly reduced when compared to the DOX group by − 43.51% in post-E group and by − 64.44% in CE group with no significant reduction in pre-E group. In contrast, skeletal tissue catalase activity considerably increased in the pre-E, post-E, and CE groups compared to the DOX group by 40.20%, 75.65%, and 99.72%, respectively. In a model of sepsis-induced skeletal myopathy, Coelho et al. [48] reported the beneficial effect of exercise in reducing oxidative stress and increasing antioxidant defense mechanisms.

Despite improvements in all oxidative stress markers, the data did not demonstrate full recovery. A significant difference still existed between both pre-E and post-E groups when compared to corresponding control values. But apparently, our results in CE group almost reached their corresponding values in control group with no significant difference. CE showed better significant antioxidant effect relative to both pre-E and post-E groups. Moreover, results indicate that the antioxidant effect of post-E is more beneficial than pre-E.

Exercise’s anti-inflammatory effects have been proposed as one of the processes by which it induces skeletal protection. Skeletal muscle TNF-α level in the present study’s pre-E, post-E, and CE groups was considerably lower by − 32.91%, − 46.91%, and − 52.23% respectively relative to DOX group. TNF-α was significantly improved in pre-E, post-E, and CE group but did not show complete recovery relative to parallel values in control group.

Calegari et al.’s [49] hypothesis was that exercise therapy modifies inflammatory mediators in a model of skeletal muscle myopathy by enhancing IL-10/TNF-α cytokine imbalance to shed more light on how exercise caused an anti-inflammatory effect.

According to Pedersen and Hoffman-Goetz [50], IL-6 is the first cytokine to be released into the bloodstream then declines after exercise followed by IL-10 increase as part of the activation of the interleukin cascade. As an anti-inflammatory cytokine, IL-6 suppresses the release of TNF-α induced by lipopolysaccharide (LPS) and stimulates the production of IL-10, which suppresses the release of chemokines and proinflammatory cytokines by various cells [51]. Furthermore, exercise triggers the release of cortisol, noradrenaline, and adrenaline, all of which can lessen the production of pro-inflammatory cytokines like IL-1β and TNF-α brought on by LPS [52]. Conversely, consistent exercise reduces the mass of white adipose tissue [53] and reduces the production of adipokines that promote inflammation, such as TNF-α [54].

Exercise increases insulin sensitivity, myofiber size, and muscle mass. Aerobic exercise typically improves insulin sensitivity and mitochondrial capacity, whereas resistance exercise is preferred to build muscle mass and strength [55]. Additionally, exercise reduces intermuscular adipose tissue infiltration and boosts muscle protein synthesis [56, 57]. Exercise training increases muscle protein synthesis and insulin sensitivity, which both contribute to increased muscle mass and decreased atrophy.

Amati et al. [58] found that consistent and regular exercise maintains high insulin sensitivity. Peterson et al. [59] found that moderate to continuous physical activity, such as walking, reduces the risk of developing metabolic syndrome, which includes insulin sensitivity. Exercise does not alter insulin signaling, which includes phosphorylating the insulin receptor, its substrate, or activating phosphatidylinositol 3-kinase. This is because exercise has been shown to increase glucose uptake in mice lacking insulin receptors [60].

The uptake of glucose by skeletal muscles is mediated by the translocation of glucose transporter 4 (Glut 4). Acute exercise-induced muscle contraction results in AMP/ATP production as well as an increase in intracellular Ca^2+^ concentration. These modifications trigger various signaling cascades, some of which probably cause the Glut 4 translocation to occur [61]. Muscle contraction requires an increase in intracellular Ca^2+^ concentration. More recently, a number of studies have shown that Ca^2+−^calmodulin signaling and Ca^2+^ calmodulin-dependent protein kinases (CaMKs) are necessary for exercise-stimulated glucose uptake. Wright et al. [62] study demonstrated that the Ca^2+/^calmodulin inhibitor KN-93 caused a decrease in glucose transport in rat skeletal muscle.

Borghouts and Keizer [63] demonstrated that exercise helps to promote cellular insulin sensitivity because it results in increased GLUT4 presence in plasma membranes and T-tubules, which keeps glucose uptake elevated for up to 120 min after exercise. Accordingly, in the current study and relative to DOX group, GLUT4 expression in skeletal muscles was significantly increased by 121.05% in the pre-E group, 284.21% in the post-E group, and 326.32% in the CE group, with significant decreases in plasma glucose, insulin, and HOMA IR respectively by − 20.89%, − 25.03%, and − 40.75% in pre-E group; − 51.89%, − 53.61%, and − 77.81% in post-E group; and − 61.04%, − 55.75%, and − 82.75% in CE group.

Although GLUT4 expression in skeletal muscles of pre-E, post-E, and CE was significantly increased relative to DOX, this increase was not sufficient to make complete recovery of insulin resistance in pre-E and post-E groups, where fasting glucose, insulin, and IR values were still higher than corresponding values in control group. As for other measured parameters in our study, CE group values showed no significant difference relative to control group. Increasing insulin sensitivity through exercise, as described by Wang et al. [64], could reduce muscle atrophy by suppressing caspase 3 activity and the proteasome proteolytic pathway that causes muscle protein degradation.

In agreement with the previous, skeletal cellular morphology as manifested from histological examination showed remarkable changes relative to DOX group. In CE and post-E groups, fibers became well-organized parallel to each other with clear transverse striations. This improvement was also detected in pre-E group but apparently not to the same level, as several pyknotic nuclei, areas of deeply acidophilic non-striated sarcoplasm, and focal loss of myofibrils were still detected.

## Conclusion

Despite DOX’s benefits as an anticancer treatment, it undoubtedly caused skeletal muscle atrophy and compromised contractile performance. However, combined exercise therapy was the most effective nonpharmacological strategy for minimizing that harmful effect relative to post-E and pre-E training strategies. So, we recommend exercise training earlier as a part of the lifestyle and even after heart failure, additionally, cancer patients should be advised to keep on mild exercise training even during chemotherapy.

## Data Availability

The datasets generated and/or analyzed during this study are available from the corresponding author on reasonable request.
